# An immunocompetent transplantable mouse model of basal cell carcinoma

**DOI:** 10.1016/j.xjidi.2026.100525

**Published:** 2026-08-12

**Authors:** Martin Wiinberg, Kristian K. Pedersen, Catharina M. Lerche, Thomas L. Andresen, Thomas Litman, Merete Haedersdal, Uffe H. Olesen

**Affiliations:** 1 Department of Health Technology, Technical University of Denmark, Kongens Lyngby, Denmark; 2 Department of Dermatology, Copenhagen University Hospital - Bispebjerg, Copenhagen, Denmark; 3 Department of Pharmacy, University of Copenhagen, Copenhagen, Denmark; 4 Department of Immunology and Microbiology, University of Copenhagen, Copenhagen, Denmark

**Keywords:** Basal cell carcinoma, Keratinocyte carcinoma, Murine cancer models, Preclinical tumor model, Tumor biology

## Abstract

Basal cell carcinoma (BCC) is the most common type of cancer, yet mouse models of BCC are limited. Existing BCC mouse models, such as the *Ptch1*^+/-^-*K14*-*CreER*^T2^*p53*^fl/fl^-tumor (genetically engineered mouse BCC [GEM-BCC]) model, rely on genetic engineering. Spontaneous tumor models are essential for studying carcinogenesis; however, the heterogeneous growth of multiple tumors requires large group sizes, making them challenging to implement in early-stage drug development. This study describes the establishment of an immunocompetent transplantable BCC (Tp-BCC) mouse model suitable for preclinical treatment evaluation. By in vivo passaging of GEM-BCC tumor cells, we established a subcutaneous Tp-BCC model. We compared the microstructural features, tumor microenvironment, and transcriptional profile of the Tp-BCC model with its parental GEM-BCC model using histology, flow cytometry, and RNA sequencing. Tp-BCC tumors displayed synchronized growth and shortened time until formation compared with GEM-BCC tumors. Tp-BCC tumors showed a micro-nodular histological structure, maintained keratin 14 expression, and had a low immune infiltration similar to GEM-BCC tumors. Hedgehog signaling pathway-related genes were similarly expressed, except for *Igf2.* The transcriptional profile of Tp-BCC tumors was more homogeneous than that of GEM-BCC tumors. This study demonstrates that the Tp-BCC model retains key features of the GEM-BCC model and provides a complementary, immunocompetent, high-throughput platform for early-stage BCC therapeutic development.

## Introduction

Annually, about 2 million people in the United States are diagnosed with basal cell carcinoma (BCC), categorizing it as the most prevalent form of cancer (Nagarajan et al, 2019). UVR is the primary risk factor for the development of BCC, and most tumors arise in sun-exposed areas. Surgical excision with safety margins is the standard of care for BCC, resulting in high cure rates. However, surgical excision may not be possible in difficult-to-treat BCC lesions, such as locally advanced, metastatic, or recurrent BCC (Peris et al, 2023). These cases rely on pharmacological treatments. In 2021, the Food and Drug Administration approved the immune checkpoint inhibitor, cemiplimab, for treating advanced BCC, which has sparked new interest in developing new immunotherapies for BCC (Davis and Lewis, 2022; Neuner et al, 2023; Stratigos et al, 2021).

Current mouse tumor models of BCC are predominantly based on genetically engineered mouse (GEM) models. These BCC models are generated by inducing mutations in Hedgehog (Hh) signaling pathway genes, such as *Ptch1*, *Smo, Gli1,* and *Shh,* to mimic the drivers of BCC carcinogenesis found in humans (Grachtchouk et al, 2000; Nilsson et al, 2000; Oro et al, 1997; Wang et al, 2011b; Xie et al, 1998). A commonly used model, developed by Ervin H. Epstein Jr’s research group, is the *Ptch1*^+/-^*K14*-*CreER*^T2^*p53*^fl/fl^ (referred to as GEM-BCC)(Wang et al, 2011b). This model has a heterozygous deletion of *Ptch1,* a tamoxifen-inducible Cre-recombinase (*Cre*) controlled by a keratin (*K*) 14 promoter, and 2 LoxP sites flanking *p53*. The Cre-Lox system deletes *p53* in K14-expressing cells, promoting genomic instability upon tamoxifen injection. Concurrent X-ray irradiation is applied to accelerate carcinogenesis. The GEM-BCC model develops multiple spontaneous tumors with histopathological and molecular genetic features similar to those of human BCC (Wang et al, 2011b).

Existing GEM models are primarily designed to study BCC carcinogenesis and are rarely used in treatment evaluation studies due to their high variation, complexity, and resource intensity compared with syngeneic cell-line-derived tumor models (Olesen et al, 2021). The first to address this challenge was Epstein Jr’s research group, who developed an allograft BCC model for the purpose of drug testing. The model was generated by subcutaneously inoculating GEM-BCC tumor cells into immunocompromised mice. Allografted tumors demonstrated similar histopathological features to the parental GEM-BCC model (Wang et al, 2011a). However, due to the compromised adaptive immune system of the recipient mouse strain, the allograft model is not applicable for the evaluation of therapeutics that interact with the immune system, such as immunotherapies.

An immunocompetent transplantable BCC model would provide a practical and higher-throughput platform for the preclinical evaluation of treatments that interact with the immune system. In this study, we aimed to develop and characterize an immunocompetent transplantable model of BCC (Tp-BCC) by in vivo passaging GEM-BCC tumor cells in immunocompetent *Ptch1*^+/-^*p53*^fl/fl^ mice. Characterization includes a comparison of the histopathology, tumor immune microenvironment, and transcriptional profile of the Tp-BCC model to the parental GEM-BCC model. The Tp-BCC model would be complementary to the GEM-BCC model and offer a practical, high-throughput platform for the preclinical evaluation of immunotherapies directed towards BCC.

## Results

### In vivo passaging reduces latency period of Tp-BCC tumors

**Figure 1:**
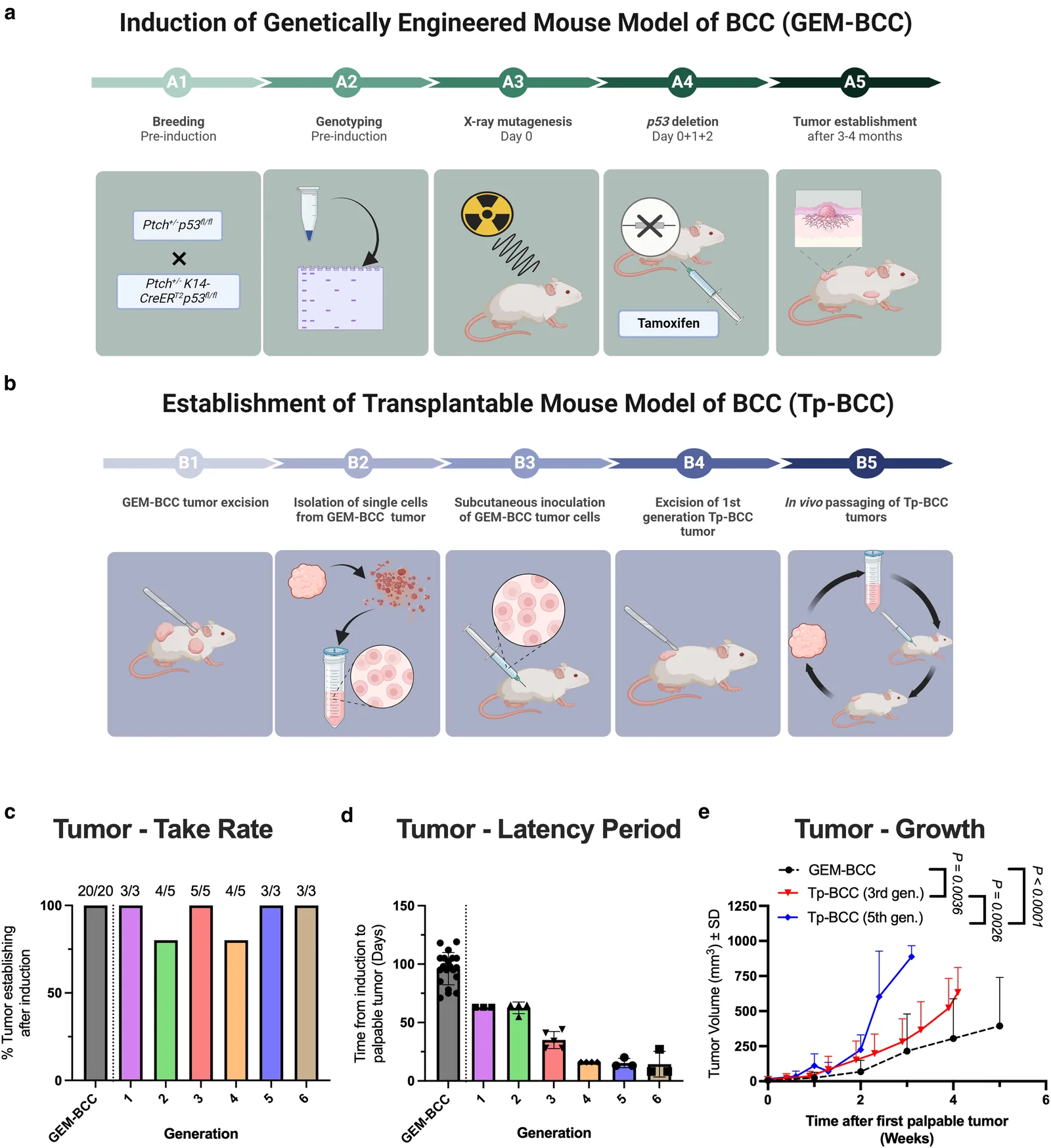
**The Tp-BCC has a shorter latency period than the GEM-BCC model.** (**a**) Induction of GEM-BCC tumors. **(b)** Establishment of Tp-BCC. **(c)** Tumor take rate in GEM-BCC and across 6 Tp-BCC generations. Numbers above bars indicate successful tumors per induction attempt. **(d)** Tumor latency period, shown as the mean number of days from induction (X-ray for GEM-BCC; inoculation for Tp-BCC) to palpable tumor. Each dot represents an individual tumor, n = 20, 3, 4, 5, 4, 3, and 3, respectively (from left to right bar). Error bars indicate ± SD. **(e)** Tumor growth illustrated as mean volume (mm^3^) over weeks post-detection. Each dot represents the mean tumor volume at the time of measurement. For GEM-BCC mice, tumor volume is calculated as the sum of all tumors on each mouse, whereas Tp-BCC mice carry a single tumor. Differences in tumor growth rates were analyzed using a linear mixed-effect model with post hoc pairwise comparisons. Error bars indicate the + SD. BCC, basal cell carcinoma; GEM-BCC, genetically engineered mouse BCC; Gen, generation; Tp-BCC, transplantable BCC.

We established Tp-BCC tumors by engrafting BCC cells originating from a GEM-BCC mouse model into non-tumor-bearing mice. The Tp-BCC tumor cells could be passaged in vivo on the GEM-BCC background strain (*Ptch1*^*+/-*^*p53*^*fl/fl*^) for at least 13 generations (Figure 1a and b). Eighty to 100% of inoculated mice in the Tp-BCC group developed tumors within the monitoring period (Figure 1c). The average time from induction to palpable tumor in GEM-BCC mice was 96 ± 14 days. In the Tp-BCC model, this latency period decreased from 63 ± 0 days in the first generation (n = 3) to 14 ± 11 days in the sixth generation (n = 3) and plateaued after the fourth generation (Figure 1d). In addition, the growth rate of Tp-BCC tumors was higher and varied less than that of tumors from the GEM-BCC model. The tumor volume of the fifth-generation group reached 800 mm^3^ 3 weeks after the tumors were palpable and grew faster than tumors in the third-generation group (*P =* .0026, Figure 1e). Cryopreserved Tp-BCC tumor cells successfully engrafted and formed tumors after thawing.

### Tp-BCC tumors maintain K14 expression

**Figure 2:**
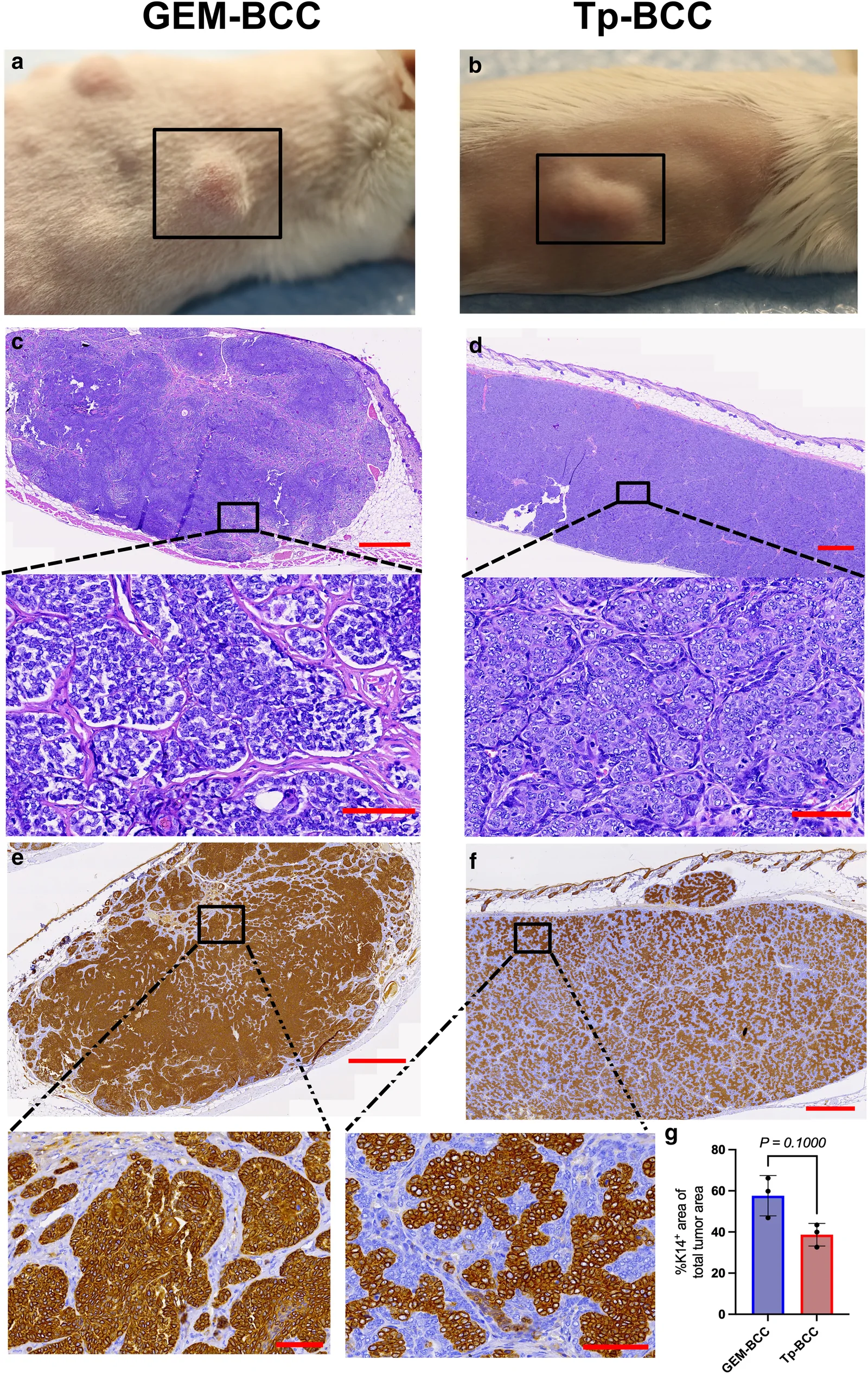
**Transplanted BCC tumors (13^th^ generation) grow in subcutaneous tissue and maintain the BCC lineage marker K14 at a reduced level.** Photographs of tumors in **(a)** GEM-BCC and **(b)** Tp-BCC models. Representative histology images of **(c)** GEM-BCC and **(d)** Tp-BCC (upper: whole slide, lower: zoomed). **(e and f)** Representative K14-DAB and hematoxylin-stained immunohistochemistry images for GEM-BCC and Tp-BCC (upper: whole slide, lower: zoomed). **(g)** Mean percentage of K14-positive area in GEM-BCC (n = 3) and Tp-BCC (n = 3). Error bars indicate ± SD. Scale bars: 600 μm (whole slides), 75 μm (zoomed). Mann-Whitney U test was used for statistical analysis. BCC, basal cell carcinoma; GEM-BCC, genetically engineered mouse BCC; K14, keratin 14; Tp-BCC, transplantable BCC.

Tumors in both models appeared superficially, making them easy to monitor (Figure 2a and b). Histological examination of 13^th^ generation Tp-BCC tumors showed tumor formation in the subcutis below the panniculus carnosus, while GEM-BCC tumors developed in the dermis. Both Tp-BCC and GEM-BCC tumors exhibited micronodular structures. These structures were most well-defined in GEM-BCC, with peripheral palisading basaloid cells, whereas Tp-BCC displayed a more chaotic structure without palisading cells. Tp-BCC tumors had a more homogeneous appearance characterized by a higher cell density with fewer extracellular matrix fibers compared with GEM-BCC tumors (Figure 2c and d).

K14 is expressed in basal cells and acts as a BCC tumor cell lineage marker in the GEM-BCC model, as *p53* is selectively deleted in K14-expressing basal cells (Wang et al, 2011b). K14 staining revealed that Tp-BCC tumors maintained K14 expression, although at a lower level than in the GEM-BCC tumors, and that most BCC tumor cells were K14-positive, while stromal cells were negative (Figure 2e and f). Quantification of the K14-positive area in the tumors showed that Tp-BCC tumors had a lower average K14 protein expression compared with GEM-BCC tumors, although this difference was not significant *(P* = .1000, Figure 2g).

### Tp-BCC tumors exhibit comparable tumor immune microenvironment

**Figure 3:**
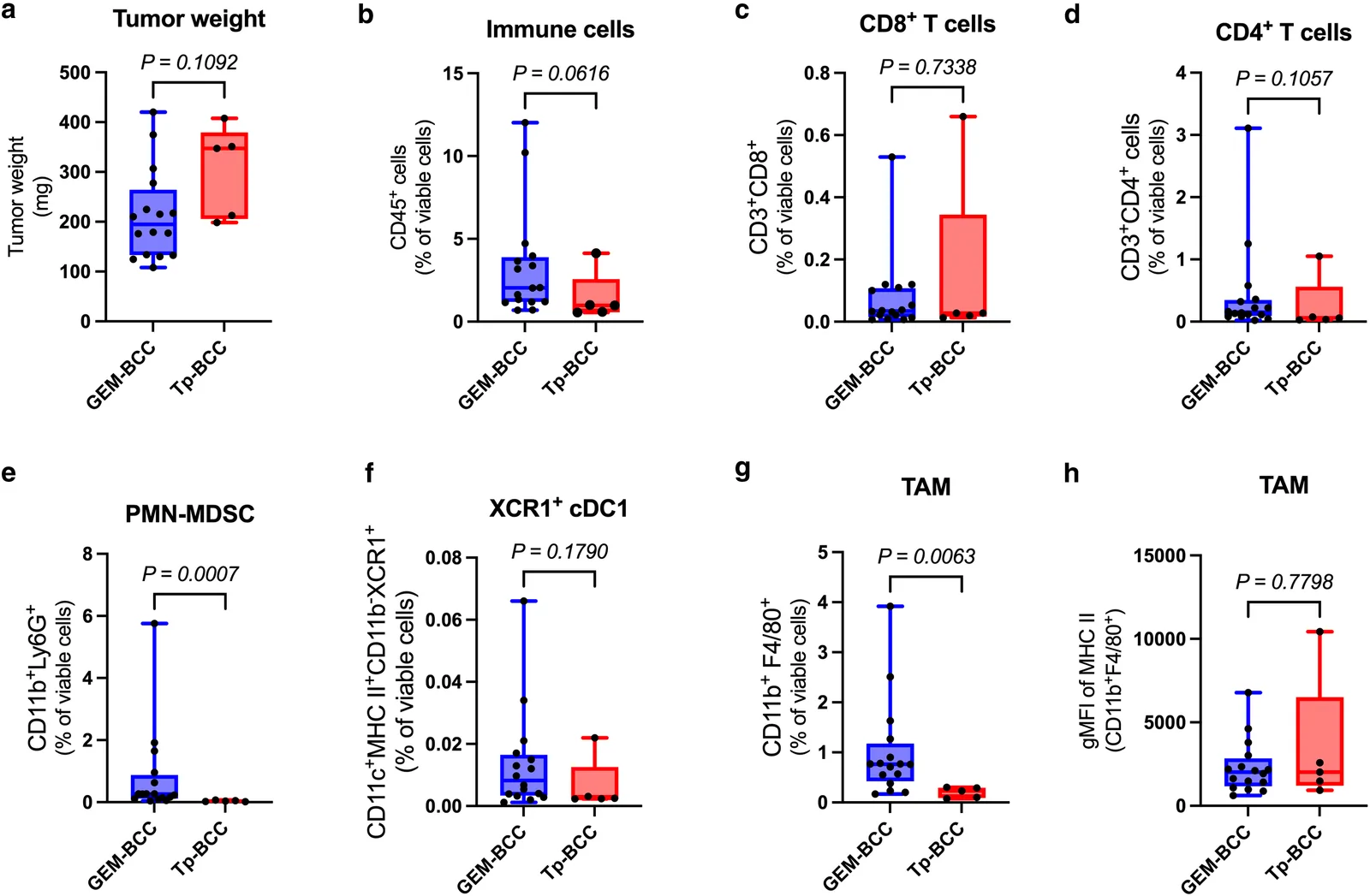
**The immune cell composition in transplanted BCC tumors is broadly similar to its parental tumor type.** (**a**) Tumor weight (mg) in GEM-BCC (n = 16) and Tp-BCC (n = 5) tumors. Percentage of **(b)** immune cells (CD45+), **(c)** CD8^+^ T cells, **(d)** CD4^+^ T cells, **(e)** PMN-MDSCs (CD11b+Ly6G^+^), **(f)** XCR1^+^ cDC1s (CD11c^+^MHCII^+^CD11b^-^XCR1^+^), and **(g)** TAMs (F4/80^+^ CD11b^+^) of all viable cells in tumor. **(h)** gMFI of MHCII on TAMs. Mann-Whitney U test was used for statistical analysis. BCC, basal cell carcinoma; cDC1, type I conventional dendritic cells; GEM-BCC, genetically engineered mouse BCC; gMFI, geometric mean fluorescence intensity; MHCII, major histocompatibility complex II; PMN-MDSC, polymorphonuclear-myeloid-derived suppressor cells; TAM, tumor-associated macrophages; Tp-BCC, transplantable BCC.

Mouse tumor models have distinct immune cell composition that influences their responsiveness to therapy (Carretta et al, 2024; Sato et al, 2021; Yu et al, 2018). We compared key immune cell types in Tp-BCC and GEM-BCC tumors of equal weight (*P* = .1092, Figure 3a). Both BCC tumor models demonstrated low immune infiltration, with immune cells constituting less than 5% of total viable cells (*P* = .0616, Figure 3b). The infiltration of CD4^+^ and CD8^+^ T cells was comparable between the models (*P* = .7338, *P =* .1057 respectively) (Figure 3c and d). GEM-BCC tumors showed an elevated percentage of polymorphonuclear-myeloid-derived suppressor cells with high variation between the tumors (*P* = .0007, Figure 3e). No significant differences were observed in XCR1^+^ type 1 conventional dendritic cells (XCR1^+^ cDC1) (*P* = .1790, Figure 3f), whereas Tp-BCC tumors had a decreased percentage of tumor-associated macrophages (*P =* .0063, Figure 3g). Further analysis of tumor-associated macrophages showed similar expression levels of major histocompatibility complex II (*P* = .7798, Figure 3h), a marker of the antitumor macrophage phenotype (Christofides et al, 2022).

### Comparable transcriptional profiles of BCC related and tumor-promoting genes

**Figure 4:**
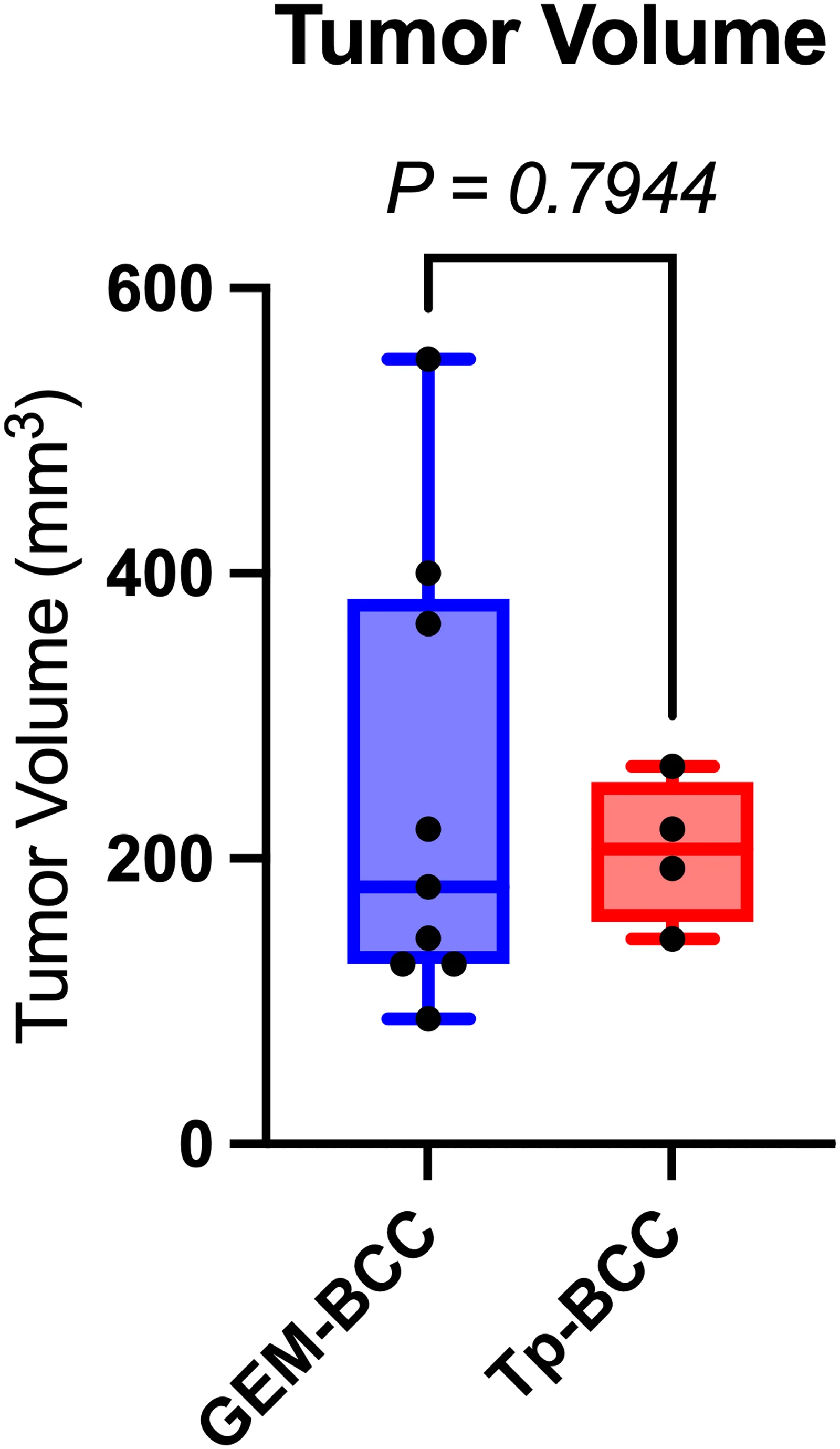
**Tumor volume (mm^3^) of RNA sequenced tumors.** Tp-BCC (n = 4) and GEM-BCC tumors (n = 9). Mann-Whitney U test was used for statistical testing. BCC, basal cell carcinoma; GEM-BCC, genetically engineered mouse BCC; Tp-BCC, transplantable BCC.

**Figure 5:**
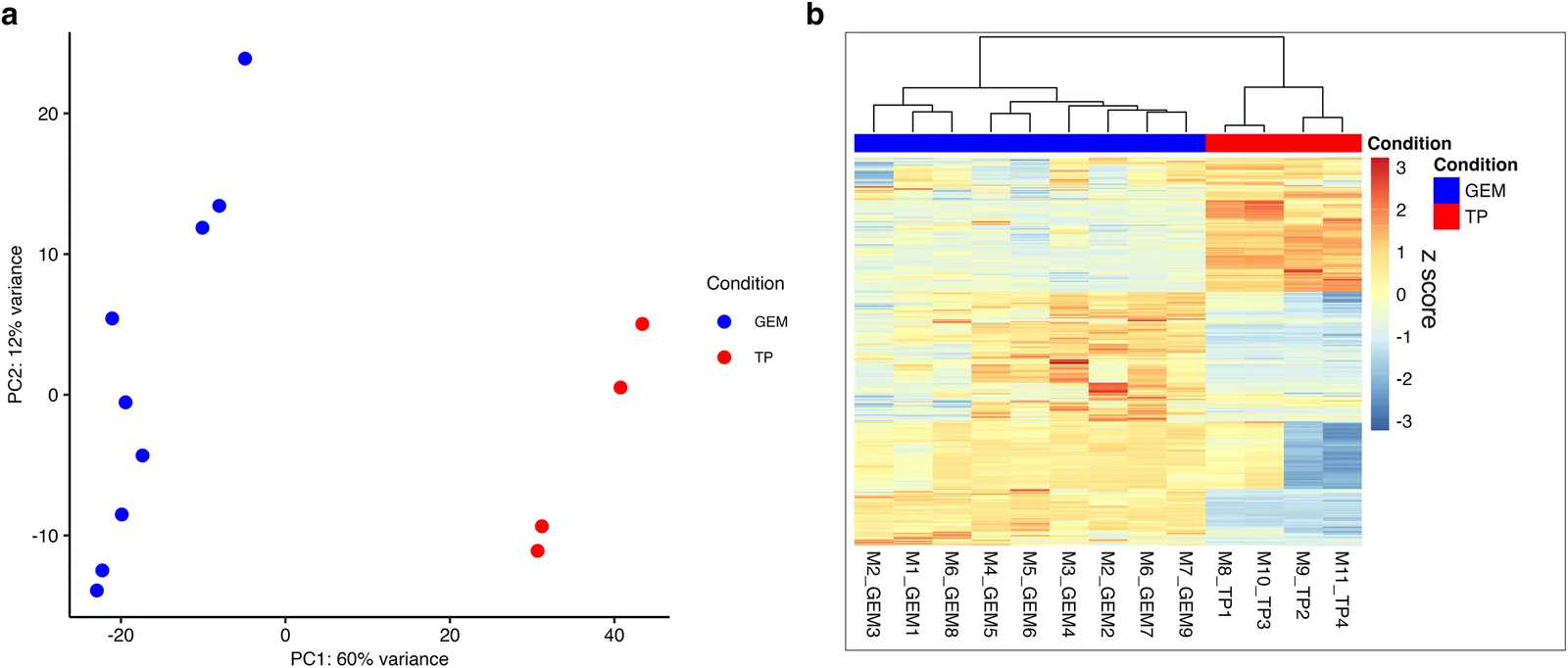
**Principal component analysis and unsupervised hierarchical clustering of Tp-BCC and GEM-BCC tumors based on the 500 most variable genes.** (**a**) PCA of GEM-BCC (n = 9) and Tp-BCC (n = 4) tumors based on variance-stabilized transformed read counts from the 500 genes with the most variance. **(b)** Heatmap and unsupervised hierarchical clustering based on variance-stabilized transformed read counts from the 500 genes with the most variance of GEM-BCC and Tp-BCC tumors. Z-scores are scaled by row. Individual mouse and tumor identification are labeled for each sample. BCC, basal cell carcinoma; GEM-BCC, genetically engineered mouse BCC; PCA, principal component analysis; Tp-BCC, transplantable BCC.

We performed RNA sequencing to compare the transcriptional profiles of volume-matched Tp-BCC (n = 4) and GEM-BCC tumors (n = 9) (Figure 4). Principal Component Analysis based on the 500 most variable genes clearly separated the 2 tumor types into distinct clusters (Figure 5a). A heatmap based on the same 500 genes supported this observation and revealed a more homogeneous expression pattern in Tp-BCC tumors compared with the transcriptional heterogeneity observed in GEM-BCC tumors (Figure 5b). When comparing the 2 groups, we identified 678 differentially expressed genes.

**Figure 6:**
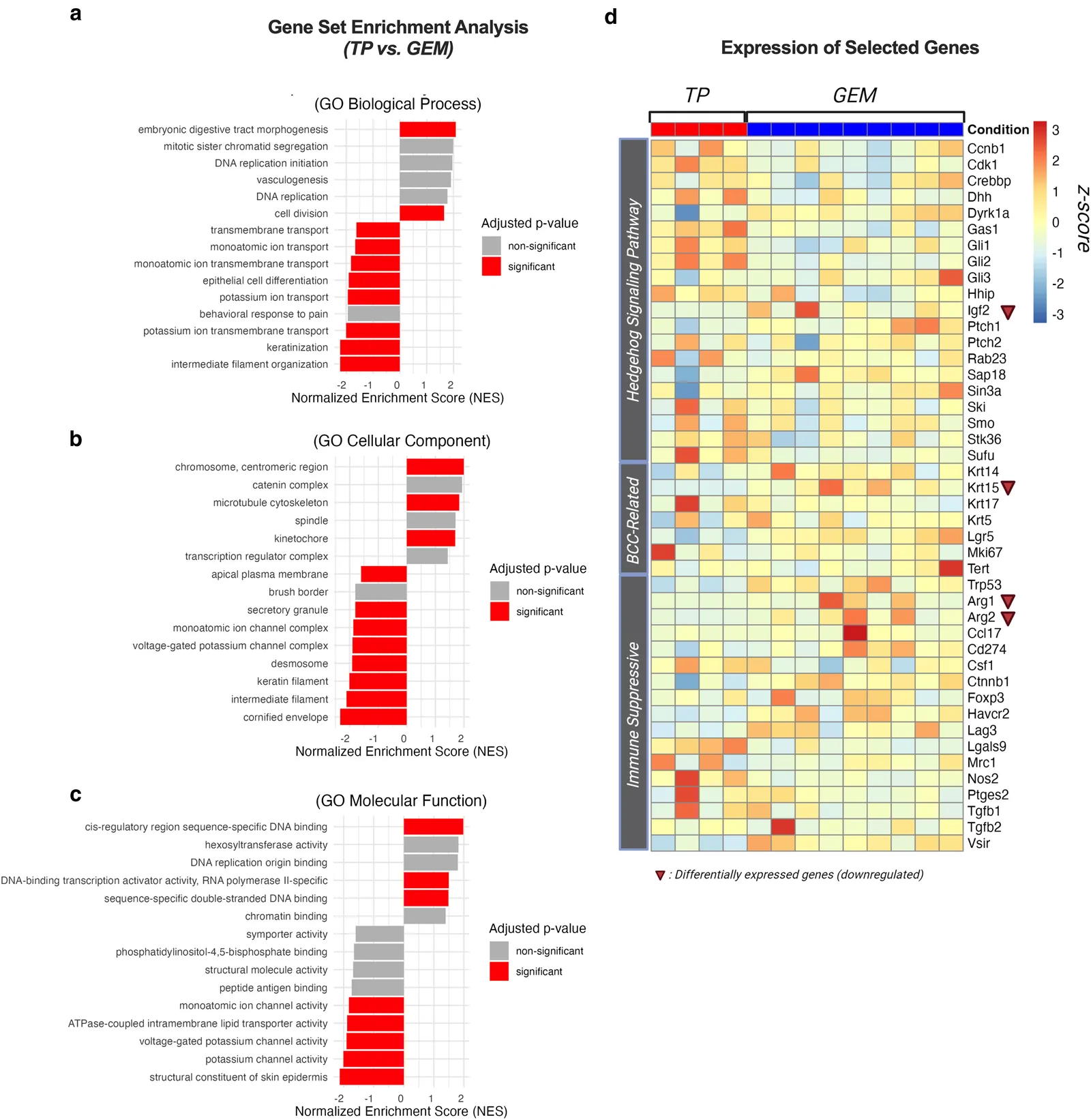
**Transcriptional analysis of BCC tumors revealed broadly similar expression profiles of tumor relevant genes.** (**a–c**) Top 15 enriched pathways from Gene set enrichment analysis using GO domains: **(a)** Biological Process, **(b)** Cellular Component, and **(c)** Molecular Function, comparing TP BCC and GEM BCC tumors. Bars represent NES; red indicates significant pathways (Adjusted *P* < .05), grey indicates non-significant pathways (Adjusted *P* > .05). **(d)** Heatmap of normalized (z-score) counts for genes related to Hedgehog signaling, BCC tumor-related, and immune suppressive genes in TP and GEM tumors. Red triangles mark downregulated genes in TP tumors (Adjusted *P* < .05, absolute log_2_ fold change (TP/GEM) >2). BCC, basal cell carcinoma; GEM, genetically engineered mouse BCC; GO, gene ontology; NES, normalized enrichment scores; TP, transplanted BCC.

To explore the functional implications of the transcriptional differences between the 2 tumor models, we performed gene set enrichment analysis using Gene Ontology (GO) terms, including biological processes, cellular components, and molecular functions. The gene set enrichment analysis revealed that gene sets associated with cell division were upregulated in Tp-BCC tumors, whereas genes associated with epithelial cell differentiation and keratinization were downregulated (Figure 6a–c). We also evaluated the expression of individual genes related to Hh signaling, other BCC-related genes, and immunosuppression (Figure 6d). Among the Hh pathway genes, only *Igf2* was differentially expressed in Tp-BCC compared with GEM-BCC. Of the BCC-related genes, *Krt15*, encoding K15, was downregulated in Tp-BCC. For immune suppressive genes, only *Arg1* and *Arg2* showed differential expression between the 2 tumor models (Figure 6d). Detailed log_2_ fold changes and adjusted *P*-values for all selected genes are listed in Table 1.

**Table 1:** List of Individual Genes Shown in Figure 6d

Gene	Log_2_FC (TP/GEM)	*P*-adj	Protein Name
**Hedgehog Signaling Pathway-related genes**			
*Ccnb1*	0.35	.2989	Cyclin B1
*Cdk1*	0.84	.0053	Cyclin-dependent kinase 1
*Crebbp*	0.17	.6668	CREB-binding protein
*Dhh*	0.49	.1783	Desert hedgehog protein
*Dyrk1a*	−0.41	.0734	Dual specificity tyrosine-phosphorylation-regulated kinase 1A
*Gas1*	0.82	.0042	Growth arrest-specific 1
*Gli1*	0.38	.1191	Zinc finger protein Gli1
*Gli2*	1.05	.0001	Zinc finger protein Gli2
*Gli3*	−0.31	.3714	Zinc finger protein Gli3
*Hhip*	0.51	.1132	Hedgehog-interacting protein
***Igf2***	**−5.90**	**< .0001**	**Insulin-like growth factor 2**
*Ihh*	N/A	N/A	Indian hedgehog protein
*Ptch1*	−0.64	.0339	Patched 1
*Ptch2*	0.12	.7262	Patched 2
*Rab23*	0.21	.4801	Ras-related protein Rab-23
*Sap18*	−0.46	.0355	Sin3A-associated protein 18
*Shh*	N/A	N/A	Sonic hedgehog protein
*Sin3a*	−0.28	.3668	SIN3 Transcription Regulator Family Member A
*Ski*	0.24	.4181	Protein homolog of avian sarcoma viral (v-ski) oncogene
*Smo*	0.08	.7704	Smoothened, frizzled class receptor
*Stk36*	0.25	.4215	Serine/threonine-protein kinase 36
*Sufu*	0.69	.0094	Suppressor of fused homolog
**Other BCC Tumor-related Genes**			
*Krt14*	−0.45	.1492	Keratin 14
***Krt15***	−**3.91**	**< .0001**	**Keratin 15**
*Krt17*	0.41	.1375	Keratin 17
*Krt5*	−0.15	.6565	Keratin 5
*Lgr5*	−1.29	.0002	Leucine-rich repeat-containing G-protein coupled receptor 5
*Mki67*	0.33	.3825	Marker of proliferation, Ki-67
*Tert*	−0.13	.7388	Telomerase reverse transcriptase
*Trp53*	−0.72	.0023	Tumor protein p53
*Continued*			
**Immune Suppressive Genes**			
***Arg1***	**−2.17**	**.0122**	**Arginase-1**
***Arg2***	**−2.39**	**.0003**	**Arginase-2**
*Ccl17*	−0.68	.1257	C-C chemokine ligand 17
*Cd274*	−0.58	.1288	Programmed cell death 1 ligand 1
*Csf1*	0.33	.2440	Macrophage colony-stimulating factor 1
*Ctla4*	N/A	N/A	Cytotoxic T-lymphocyte associated protein 4
*Ctnnb1*	−0.62	.0163	Catenin beta-1
*Foxp3*	−0.11	.7850	Forkhead box P3
*Havcr2*	−1.68	.0088	Hepatitis A virus cellular receptor 2
*Ido1*	N/A	N/A	Indoleamine 2,3-dioxygenase 1
*Ido2*	N/A	N/A	Indoleamine 2,3-dioxygenase 2
*Il10*	N/A	N/A	Interleukin-10
*Il4*	N/A	N/A	Interleukin-4
*Lag3*	−0.65	.0506	Lymphocyte activation gene 3
*Lgals9*	1.12	.0003	Galectin-9
*Mrc1*	0.27	.4658	Mannose receptor C-type 1
*Nos2*	1.25	.0005	Nitric oxide synthase, inducible
*Pdcd1*	N/A	N/A	Programmed cell death protein 1
*Pdcd1lg2*	N/A	N/A	Programmed cell death 1 ligand 2
*Ptges2*	N/A	N/A	Prostaglandin E synthase 2
*Tgfb1*	0.27	.4029	Transforming growth factor beta-1
*Tgfb2*	−0.07	.8793	Transforming growth factor beta-2
*Tigit*	N/A	N/A	T cell immunoreceptor with immunoglobulin and ITIM domain
*Vctn1*	N/A	N/A	V-set domain-containing T-cell activation inhibitor 1
*Vsir*	−1.28	< .0001	V-set immunoregulatory receptor

Abbreviations: BCC, basal cell carcinoma; GEM-BCC, genetically engineered mouse BCC; Log_2_FC, Log_2_ fold change; *P-*adj, Adjusted *P*-value; Tp-BCC, transplantable BCC.

Genes in bold were differentially expressed in Tp-BCC tumors compared with GEM-BCC tumors (TP/GEM) (*P-*adj < .05, absolute Log_2_ fold change > 2). N/A indicates genes with too low read counts to be included in analysis.

## Discussion

In this study, we established a Tp-BCC tumor model in immunocompetent mice by transplanting tumor cells derived from the GEM-BCC model. The Tp-BCC model retains key features of the GEM-BCC model while providing practical advantages for preclinical studies, including synchronized tumor growth, reduced variability, and high-throughput testing.

The Tp-BCC showed an increased tumor growth rate and a reduced latency period from the first to the fifth generation of in vivo passaging. The increased growth rate is consistent with previous findings in the allograft BCC model established by Epstein Jr’s research group (Wang et al, 2011b). Because the same number of cells was inoculated in each passage, serial in vivo passaging likely caused a natural selective pressure favoring highly proliferative tumor cells and thereby generated a homogeneous tumor cell population over time. Tp-BCC tumors exhibited a more homogeneous histological organization than GEM-BCC tumors. This is potentially caused by an increased ratio of tumor cells to stromal cells in the inoculum over passaging generations.

The selective pressure is further supported by the decreased K14 protein expression observed in the Tp-BCC tumors at the 13^th^ generation of in vivo passaging compared with GEM-BCC tumors, although this difference did not reach statistical significance. Importantly, Tp-BCC tumors retained detectable K14 expression at this late passage, which indicates partial maintenance of basal epithelial characteristics despite extensive passaging. Because K14 expression was assessed only at the 13^th^ generation and not across earlier or later passages, the long-term persistence cannot be determined from the current study. Nevertheless, the reduced K14 expression suggests that while K14 is important for tumor initiation in the GEM-BCC model, it may be less essential for continued tumor growth following subcutaneous transplantation and therefore becomes downregulated during in vivo passaging. This interpretation is supported by the reduced *Krt15* gene expression observed in the transcriptional profiling of Tp-BCC tumors. K15 is a marker of basal stem cells in the stratified epithelial tissue and has been reported to be upregulated in nodular BCCs and to play an important role in maintaining epithelial integrity (Jih et al, 1999; Kurzen et al, 2001; Lloyd et al, 1995; Porter et al, 2000). Together, reduced K14 and *Krt15* expression, along with downregulation of genes associated with epithelial cell differentiation and structural components of the epidermis, suggests that Tp-BCC tumor cells undergo partial dedifferentiation from their basal epithelial lineage, potentially as a consequence of serial transplantation in the subcutaneous tissue.

When evaluating therapeutic responses, it is important to consider the role of the immune system, as previous studies have shown that the immune system can affect the efficacy of conventional treatments such as chemotherapy and radiation therapy (Apetoh et al, 2007; Nowak et al, 2003; Zitvogel et al, 2008). A key advantage of the Tp-BCC model is its establishment in immunocompetent mice, making it suitable for studying treatment responses in the context of an intact immune system, whereas the allografted BCC model developed by Epstein Jr’s group relies on immunocompromised mice. The immune cell composition within the tumor microenvironment was investigated because it is known to influence treatment outcome in solid tumor models (Sato et al, 2021; Yu et al, 2018). Here, we found that the Tp-BCC tumors were poorly immune infiltrated by immune cells, which comprised less than 5% of all viable cells found in the tumor, similar to the GEM-BCC. Although the overall immune composition was broadly comparable between the 2 models, Tp-BCC tended to show less intertumoral variation across immune cell populations. Tp-BCC tumors exhibited a significantly lower proportion of polymorphonuclear-myeloid-derived suppressor cells and tumor-associated macrophages compared with GEM-BCC tumors. Both cell populations are known to be contributors to an immunosuppressive environment (DeNardo and Ruffell, 2019; Raskov et al, 2022). Consistently, GEM-BCC tumors showed significantly higher expression of the immunosuppressive enzymes, Arg-1 and Arg-2, which are known to be predominantly expressed by tumor-associated macrophages and polymorphonuclear-myeloid-derived suppressor cells and have been associated with poor clinical outcomes across multiple cancer types (Ino et al, 2013; Lang et al, 2018; Pokrývková et al, 2021). The reduced Arg1 and Arg2 expression in Tp-BCC tumors therefore likely reflects the decreased abundance of these myeloid populations and suggests that the microenvironment of Tp-BCC tumors may harbor a less myeloid-driven immune suppression than that of GEM-BCC tumors.

Despite these differences, the immune infiltration of both models was low compared with commonly used syngeneic cell-line-derived transplanted models, in which immune cells often constitute between 20% and 70% of all viable cells within the tumor (Carretta et al, 2024; Jin et al, 2022; Sato et al, 2021). Both BCC models also showed low infiltration of CD8^+^ and CD4^+^ T cells compared with the widely used CT26 and MC38 cell-line-derived tumor models (Carretta et al, 2024; Sato et al, 2021; Yu et al, 2018). Consistent with our findings, human BCC has been reported to display low immune infiltration, particularly of CD8^+^ T cells (Omland et al, 2022). Reduced HLA class I expression has also been associated with low T cell infiltration in human BCC (Oka et al, 2025). Analysis of the transcriptional data did not reveal significant differences in major histocompatibility complex class I gene expression between Tp-BCC and GEM-BCC tumors. It remains unclear whether the overall low T cell infiltration observed in the murine BCC tumors is associated with reduced major histocompatibility complex class I expression relative to more immunogenic tumor models.

Global gene expression analysis identified 678 differentially expressed genes between Tp-BCC and GEM-BCC tumors. Notably, none of the Hh signaling pathway-related genes, except for *Igf2* were differentially expressed in the Tp-BCC. Although *Igf2* has been proposed as a downstream target of Hh signaling, its importance in BCC biology is unknown (Hahn et al, 2000). Importantly, the Tp-BCC tumors maintained *Gli1* expression at levels similar to those of GEM-BCC tumors. This finding suggests that the model can be used for testing drugs targeting the Hh signaling pathway. By contrast, the allografted BCC tumor model developed by Epstein Jr’s research group, displays reduced *Gli1* expression after transplantation (Wang et al, 2011a).

The fact that promising drug efficacy in preclinical cancer studies often fails to translate into human clinical trials suggests that inadequate disease modeling and limited understanding of in vivo mechanisms are key factors contributing to the translational gap (Arrowsmith and Miller, 2013; Johnson et al, 2001; Mullard, 2016). A systematic review by Gengenbacher et al found that 82% of preclinical studies include only 1 tumor model in their studies (Gengenbacher et al, 2017). We therefore propose that including complementary models of increasing biological complexity may enhance the translational value of the preclinical findings. In the therapeutic development framework, the Tp-BCC model would be particularly useful for early-stage drug candidate screening, dose optimization, and drug mechanism-of-action studies before final evaluation in the more clinically relevant GEM-BCC model. The practical advantages of the Tp-BCC model in early stages are also reflected in its higher throughput and shorter latency, with 6^th^ generation. Tp-BCC tumors becoming palpable after a mean of 14 days compared with 96 days for GEM-BCC tumors. However, because the Tp-BCC tumors form following injection of cancer cells, the model is not suited for studying the tumorigenesis of BCC, including tumor initiation and immune editing. In addition, Tp-BCC tumors develop in an ectopic subcutaneous location, which may influence antitumor immune responses. For example, intradermally implanted tumors have been reported to be more readily rejected than subcutaneous tumors because the higher density of dendritic cells in the dermis enables recruitment and antigen presentation (Bonnotte et al, 2003; Joncker et al, 2016). Although the Tp-BCC model does not model human BCC to the same extent as the GEM-BCC model, it offers important practical advantages for therapeutic evaluation, such as reduced latency, lower cost, and increased experimental throughput. Together, these features position the Tp-BCC model as a platform for early-stage therapeutic development, in a setting in which transplantable immunocompetent models are currently lacking. The Tp-BCC model should therefore be considered complementary to the GEM-BCC model rather than a replacement.

Compared with common syngeneic cell-line-derived tumor models used for the evaluation of immunotherapy, such as the CT26 and MC38, the Tp-BCC model offers a disease-relevant platform for BCC research while remaining less resource-intensive than the GEM-BCC model. However, the model also has technical limitations. The Tp-BCC has not been established as a stable in vitro cell line; however, to our knowledge, no currently available mouse BCC cell line forms tumors in immunocompetent mice. In addition, the Tp-BCC model remains dependent on serial in vivo passaging, and thus phenotypic drift over time cannot be excluded. Nevertheless, tumor cells from a Tp-BCC tumor can be cryopreserved and later inoculated into mice, with the potential of generating more than 20 new tumors from a single tumor, thereby reducing the need for continuous passaging. The Tp-BCC model is currently restricted to the mixed genetic background strain of the GEM-BCC mice, for which histocompatibility has not been determined. Determining the haplotype of the GEM-BCC background strain may help identify a commercially available mouse strain that is histocompatible with the Tp-BCC tumor cells and thereby reduce the dependence on the GEM-BCC breeding colony. However, the use of surplus mice from the GEM-BCC breeding process aligns with the *Reduction* principle of the replacement, reduction and refinement (3Rs). Further studies are also needed to determine how well treatment responses in the Tp-BCC model translate into responses in GEM-BCC tumors.

A limitation of the gene expression analysis is the potential presence of batch effects due to technical variation introduced by collecting, isolating, and sequencing RNA from Tp-BCC and GEM-BCC tumor samples in separate batches. Although such batch effects cannot be fully excluded, they would be expected to exaggerate gene expression differences between the models. However, the observed overall transcriptomic similarity between Tp-BCC and GEM-BCC was high.

In summary, we developed a mouse model of BCC, Tp-BCC, that shares key histological, tumor immune microenvironment, and transcriptional features of the well-established GEM-BCC model, while enabling faster, more synchronized, and resource-efficient preclinical studies. The Tp-BCC model is not intended to replace the GEM-BCC model but to provide a complementary model for early-stage therapeutic screening and mechanistic studies prior to validation in the more clinically relevant GEM-BCC model.

## Materials and Methods

### Animals

Immunocompetent female transgenic mice of the following genotypes, *Ptch1*^+/-^*K14*-*CreER*^T2^*p53*^fl/fl^ and *Ptch1*^+/-^*p53*^fl/fl^ were used in the study (Wang et al, 2011a). *Ptch1*^+/-^*K14*-*CreER*^T2^*p53*^fl/fl^ and *Ptch1*^+/-^*p53*^fl/fl^ mice were bred at the Department of Experimental Medicine, University of Copenhagen, and transported to the Department of Dermatology, Copenhagen University Hospital—Bispebjerg Hospital. Mice were acclimatized for at least 1 week before being included in the studies. Mice were housed at 24 °C with *ad libitum* feeding and a 12-hour light/dark cycle.

### Tumor establishment and in vivo passaging

**Figure 7:**
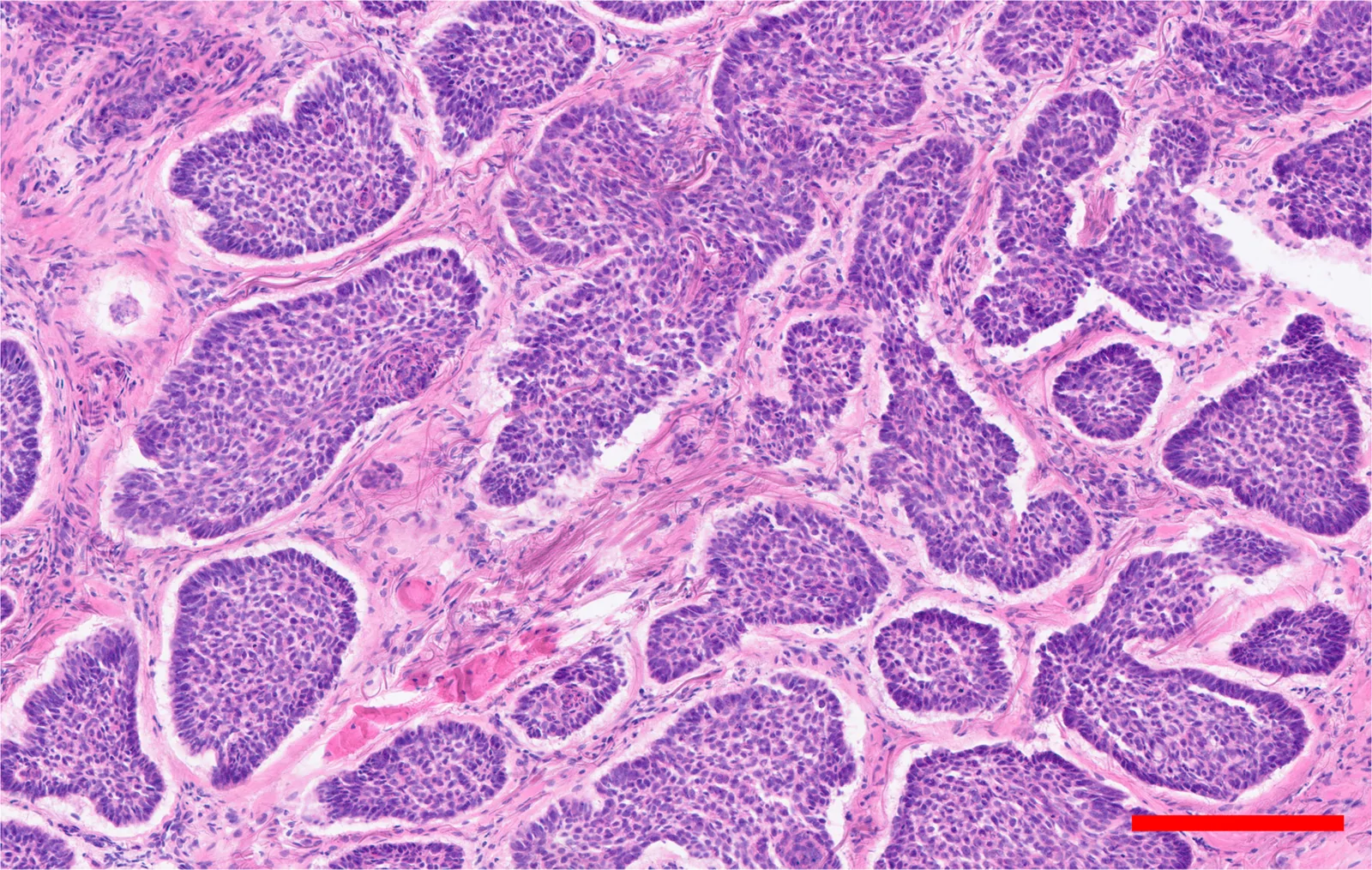
**Histology of human BCC.** An H&E-stained human BCC demonstrating characteristic basaloid tumor nests within the dermis and peripheral palisading and stromal retraction artifact. Written informed consent for research use of the tissue sample was obtained from the patient. Scale bar = 200 μm. BCC, basal cell carcinoma.

GEM-BCC tumors were induced in *Ptch1*^+/-^*K14*-*CreER*^T2^*p53*^fl/fl^ mice. Mice were X-ray irradiated on the dorsal skin (Model D3100, Gulmay Medical, Surrey, UK) with 4 Gray (50 kV for 2.05 minutes) under sedation with 0.3 ml HypNorm (fentanyl citrate 0.158 mg/ml, fluanisone 5 mg/ml, and midazolam 2.5 mg/ml). To induce the Cre-recombinase system, mice were injected intraperitoneally with 300 μg tamoxifen (Sigma-Aldrich, Munich, Germany) for 3 consecutive days. Palpable tumors were observed approximately 3 to 4 months after induction (Figure 1a). Multiple palpable tumors were observed on the dorsal skin approximately 3 to 4 months after induction (Figure 2a). The histology of GEM-BCC tumors was examined by a Mohs surgeon to confirm their resemblance to human BCC (Figure 7).

The first generation of Tp-BCC tumor model was generated by inoculating tumor cells from the parental GEM-BCC tumors, as described in Figure 1b. In short, a single GEM-BCC tumor measuring 8 mm in length and width was aseptically excised and minced using scalpels. Tumor fragments were placed in 5 ml Mouse Tumor Dissociation enzyme mix kit (Milteney Biotec, Bergisch Gladbach, Nordrhein-Westfalen, cat#130-096-730) for 1 hour in a heat chamber at 37 °C in while tubes were rotating in a Mini LabRoller tube rotator (LabNet International, Edison, New Jersey, USA). Digested tumor fragments were strained through a 70 μm filter (pluriSelect Life Science, Leipzig, Sachsen, Germany, cat#43-10070) and flushed with 4 °C sterile PBS, then centrifuged at 380 g for 5 minutes. The pellet was filtered, flushed, and centrifuged once again as described above. Cells were counted, and viability was determined using trypan blue and the electronic cell counter, Countess I (Thermo Fisher Scientific, Waltham, Massachusetts, USA). The cell concentration was diluted to 20 million viable cells per milliliter in Hank’s Balanced Salt Solution. Two million cells were then subcutaneously injected into the right flank of *Ptch1*^+/-^*p53*^fl/fl^ mice. The Tp-BCC developed a single tumor at the site of inoculation. Tp-BCC tumor cells were serially in vivo passaged by transplanting tumor cells from the previous Tp-BCC generation into a new host mouse using the approach described above (Figure 1b).

Tumors were measured with a digital caliper. Tumor volume was determined as $Length\times Width^{2}\times 0.5$. As multiple tumors may be present in the GEM-BCC model, the tumor volume of each sample was presented as the sum of all tumors present on the GEM-BCC mouse at each time point.

### Histology and image pixel quantification

Tumors were excised and fixated in 4% formalin buffer and processed with the Shandon Excelsior ES (Thermo Fisher Scientific, Waltham, Massachusetts, USA) and embedded in paraffin. 4 μm sections were cut with a Shandon Finesse Series Microtome (Thermo Fisher Scientific, Waltham, Massachusetts, USA). H&E staining was carried out by first deparaffinizing and rehydrating the tissue before staining with Mayer’s hematoxylin for 5 minutes, washed, and stained with eosin for 5 minutes. Sections were dehydrated with ethanol before mounting with Pertex Mounting Medium.

**Figure 8:**
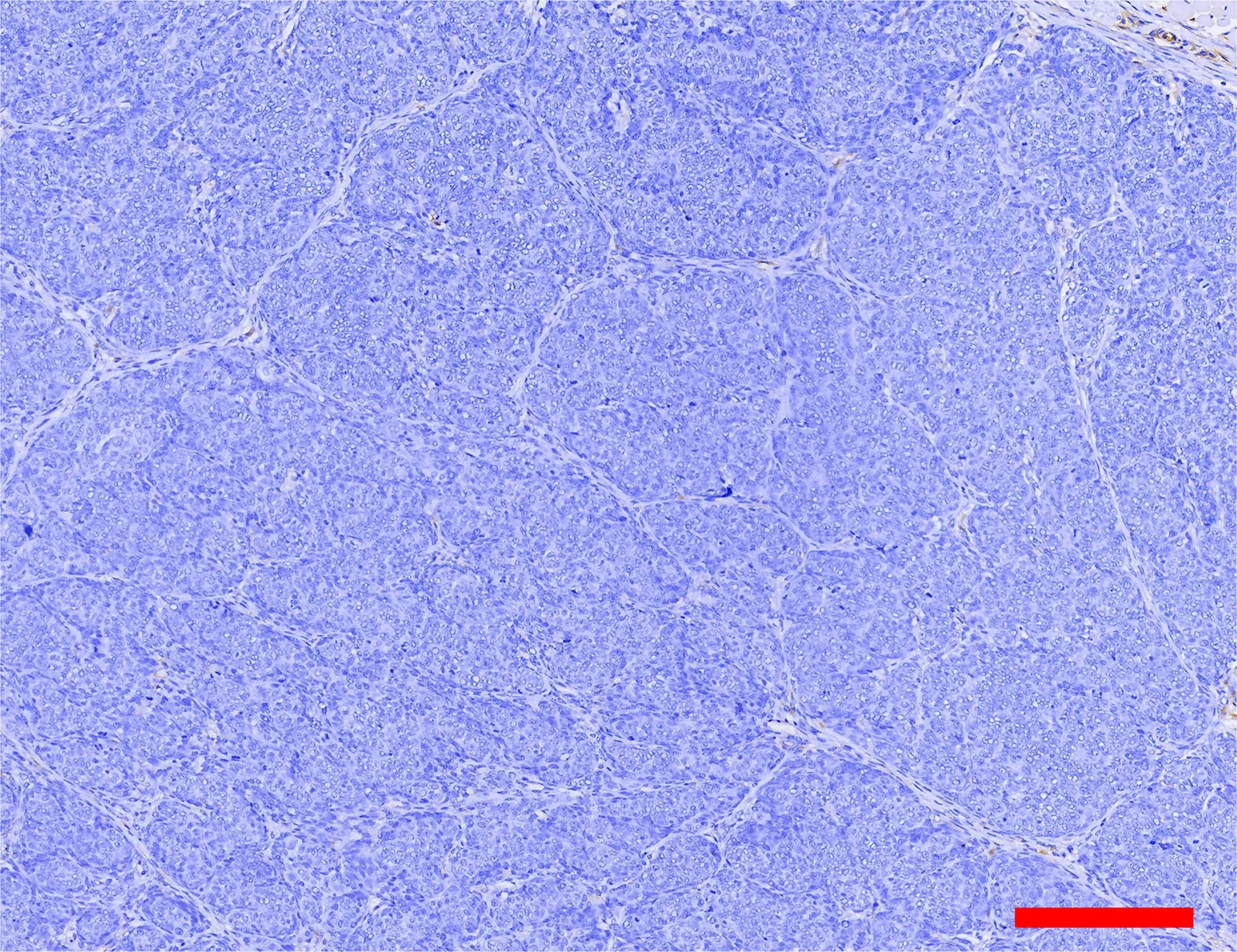
**No-primary antibody (K14) control to determine non-specific DAB+ staining.** Immunohistochemical slides stained without primary K14 antibody but only with secondary antibody prior to DAB+ staining. Scale bar = 200 μm. Abbreviations: K14: Keratin 14.

For immunohistochemistry staining, 4 μm sections were heat-induced epitope retrieved with pH 6 citrate buffer for 20 minutes at 95–100 °C. Sections were blocked with 2% BSA in tris-buffered saline for 15 minutes. An optimal antibody dilution of 1:2000 was determined by titration prior to coating with primary rabbit cytokeratin 14 antibody solution (BioLegend Incorporated, San Diego, California, USA, cat#905303) for 1 hour at room temperature. Next, sections were first exposed to peroxidase-blocking solution (Dako Real, Agilent, Glostrup, Denmark) for 10 minutes before being treated with secondary goat anti-rabbit/mouse Horseradish Peroxidase-conjugated antibody (Dako EnVision+ System, Agilent, cat#K500711-2). Stains were developed with DAB+ chromogen in substrate buffer (Dako Real EnVision+ System, Agilent) for 5 minutes and counterstained with hematoxylin for 15 seconds before dehydration and mounting with Pertex Mounting medium. No-primary antibody control showed no notable non-specific staining (Figure 8). Histology slides were digitally scanned at 80× magnification using the MoticEasyScan Pro (Motic, Xiamen, Fujian, China). QuPath Software version 0.5.1(Bankhead et al, 2017) (https://github.com/qupath) was used to quantify the relative K14 positive area in tumors. The total tumor area was segmented before measurement of DAB stain positive pixels using the pixel thresholding function in QuPath Software (Table 2).

**Table 2:** Pixel Classification Parameters in QuPath Used for Segmentation and Quantitative Measurement of Keratin 14 Positive Area in Immunohistochemical Samples

Parameter	Regional Segmentation	Keratin 14 Measurement
Resolution	Full (down sample = 1.00)	Full (down sample = 1.00)
Channel	Average channels	DAB
Prefilter	Morphological opening	Gaussian
Smoothing sigma	1	0.1
Threshold	230	0.4
Above threshold	Region	Positive
Below	Ignore	Negative
Region	Everywhere	Everywhere

### Flow cytometry

A single-cell suspension from BCC tumors between 100 and 420 mg (Figure 3a) was obtained using the same approach as for tumor establishment. The samples were subsequently stained. In short, Fc receptors were blocked with purified rat anti-mouse CD16/CD32 (BD Biosciences Dickinson, San Jose, California, USA, cat#553141), and surface staining was performed for 30 minutes with the antibody cocktail provided in Table 3. Samples were acquired on the LSRFortessa X-20 Flow Cytometer (BD Biosciences). Single antibody staining of Ultra-Comp eBeads Plus Compensation Beads (Thermo Fisher Scientific, cat#01-3333) and viability dye staining of ArC Amine Reactive Compensation Beads (Thermo Fisher Scientific, cat#A10346) were used to determine spectral spillover using FlowJo Software version 10.10 (BD Biosciences).

**Table 3:** List of Antibodies and Viability Dye Used for Flow Cytometry Analysis of Tumors

Antigen	Clone	Fluorophore	Dilution	Supplier	Cat#.
CD45	30-F11	BUV395	1:200	BD Biosciences	564279
CD8a	53-6.7	BUV737	1:100	BD Biosciences	612759
XCR1	ZET	BV421	1:200	BioLegend	148216
CD11b	M1/70	BV480	1:400	BD Biosciences	566149
CD4	RM4-5	BV605	1:200	BD Biosciences	563151
CD11c	HL3	BV786	1:100	BD Biosciences	563735
I-A/I-E (MHCII)	M5/114.15.2	FITC	1:200	BioLegend	107606
CD3	145-2C11	PE	1:100	BD Biosciences	553064
F4/80	BM8	PE-Cy7	1:400	Thermo Fisher Scientific	25-4801-82
Ly6G	1A8	APC	1:200	BioLegend	127613
*Dead cells*^1^	N/A	eFluor 780	1:500	Thermo Fisher Scientific	65-0865

Abbreviation: Cat#: Catalogue number of item.

1 Viability dye used to discriminate dead cells from viable cells.

**Figure 9:**
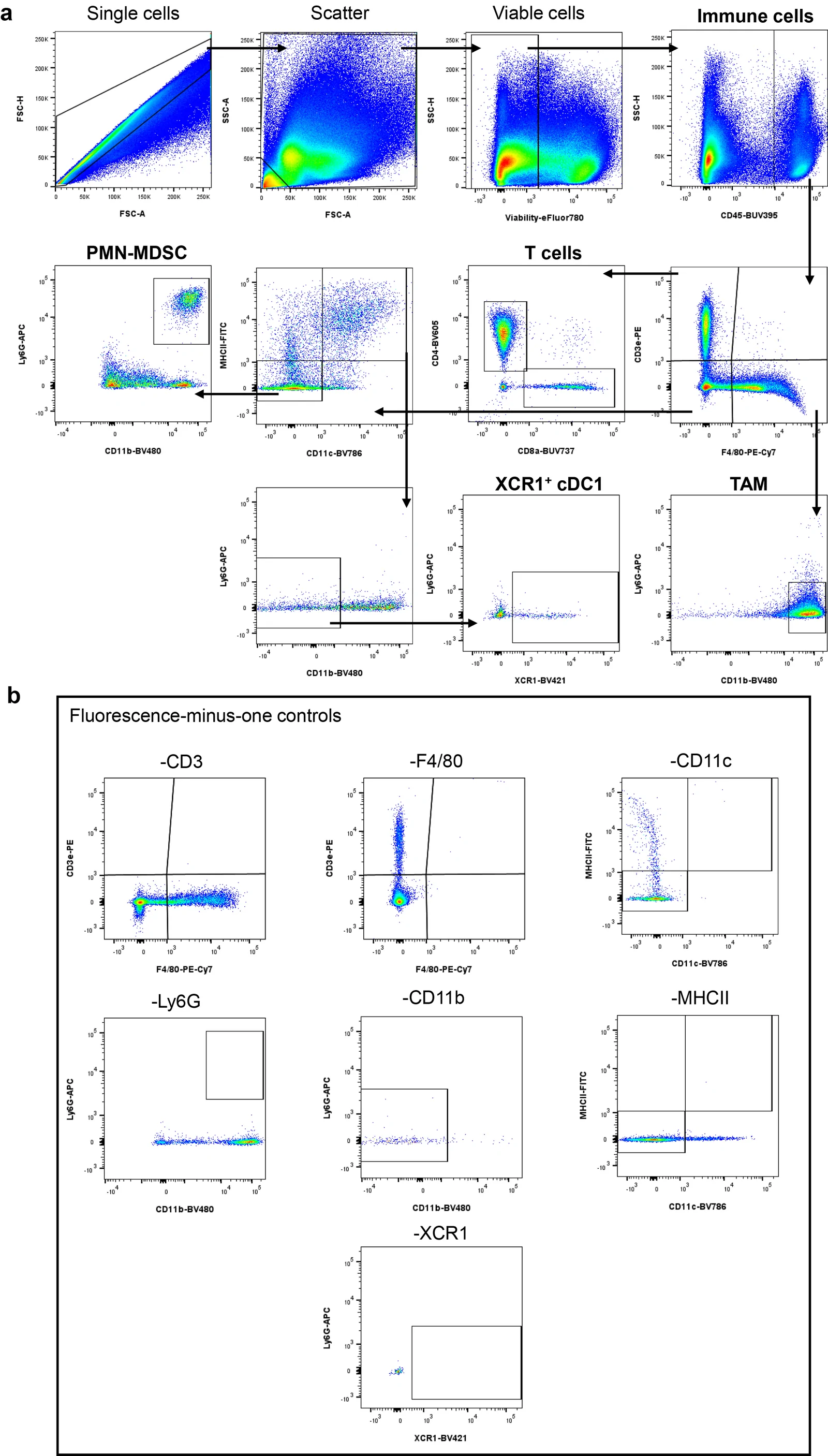
**Gating strategy of flow cytometry data.** (**a**) Gating of flow cytometry events in FlowJo software. Plot titles indicate the gated cell types **(b)** FMO controls. Plot titles indicate the missing antibody. Axis labels indicate target antigen and fluorophore. cDC1, type I conventional dendritic cells; FMO, fluorescence-minus-one; K14, keratin 14; PMN-MDSC, polymorphonuclear-myeloid-derived suppressor cells; TAM, tumor-associated macrophages.

Analysis was performed using FlowJo Software version 10.10. Figure 9 presents the gating strategy and fluorescence minus-one samples. The represented tumor data were pooled from 4 experiments conducted over 4 months. Each experiment was compensated and gated individually with fluorescence minus-one samples. In addition, Sphero Rainbow Calibration Particles (8 peaks, BD Biosciences, cat#559123) were used to calibrate the photomultiplier tube detectors of every channel between experiments to enable comparison of the geometric mean fluorescent intensity.

### RNA extraction, library preparation, and sequencing

Tumors were excised, cut into smaller fragments, preserved in RNAlater solution (Thermo Fisher Scientific, cat#AM7021), and stored at −20 °C until further processing. RNA was isolated using TRIzol (Thermo Fisher Scientific, cat# 15596026), homogenization was performed with the Retsch MM 400 mixer mill (Verder Scientific, Haan, Nordrhein-Westfalen, Germany) and Acid-Phenol:Chloroform (Thermo Fisher Scientific, cat#AM9722). RNA was purified with the NucleoSpin RNA kit (Düren, Nordrhein-Westfalen, Germany, cat#740955.50).

Library preparation and RNA sequencing was performed by BGI Genomics (Shenzhen, China). Oligo(dT)-attached magnetic beads were used for mRNA enrichment before poly(A) tail fragmentation and reverse transcription. cDNA double strands were end-paired, 5′phosphorylated, and ligated with a bubble-shaped adapter and then amplified with PCR. Constructed libraries were quality-checked, and samples with an RNA quality number (RQN) >10 were sequenced on the DNBSEQ-G400 platform (Shenzhen, China) with a 150 paired-end read length. The SOAPnuke package version 1.5.6 (parameters: -l 15 -q 0.2 -n 0.0001 -G) was used to clean raw reads. Reads were aligned to the National Center for Biotechnology Information *Mus musculus* genome (Mus_musculus_10090.NCBI.GCF_000001635.27_GRCm39.v2201) using Bowtie version 2.3.4.3 (parameters: -q –sensitive –dpad 0 –gbar 99999999 –mp 1,1 –np 1 –score-min L,0,-0.1 -l 1 -X 1000 –no-mixed –no-discordant -p 8 -k 20 -N 1) and quantified using RSEM version 1.3.1 (-p 8 –forward-prob 0).

### Gene expression analysis

Genes with fewer than 10 read counts in 4 or more samples were not included in the analysis. Differential expression analysis was performed using the DESeq2 R package with Benjamini-Hochberg correction to adjust *P*-values for multiple testing. Fold changes were shrunk using the Adaptive Shrinkage R package (Stephens 2017). Genes with an adjusted *P*-value < .05 and log_2_ fold change >2 were considered significantly differentially expressed genes. gene set enrichment analysis was conducted using the fgsea R package to analyze functional enrichment of gene sets from the GO terms: Biological Process (GO: 0008150), Cellular Components (GO:0005575), and Molecular Function (GO: 0003674). Read counts of all genes were normalized using the median of ratios method provided by the DESeq2 package prior to generating the heatmap of selected genes in Figure 6d using the pheatmap R package. Genes related to the Hh signaling pathway were obtained from the WikiPathways mouse gene set (WP_HEDGEHOG_SIGNALING_PATHWAY, WP116 https://www.wikipathways.org/pathways/WP116.html). BCC-related and immunosuppressive genes presented in Figure 6d and Table 1 were selected by the authors prior to analysis. Read counts were normalized using variance stabilizing transformation before Principal Component Analysis with the prcomp R package, as presented in Figure 5a. A heatmap and unsupervised hierarchical clustering of samples were done using the pheatmap R package, as shown in Figure 5b. Figure 5 was generated using the 500 most variable genes.

### Statistical analysis

The data were visualized using Prism version 10.2 (GraphPad Software, San Diego, California, USA) for Figure 1, Figure 2, Figure 3, Figure 4 and RStudio version 2024.04.1 (R Core Team) (Vienna, Austria, https://www.R-project.org/) for Figure 1e and Figure 5, Figure 6. Graphical illustrations were created with BioRender.com. Comparisons of protein expression in histological samples and cell populations in flow cytometry analysis were made using the Mann-Whitney U test in Prism. Tumor growth in Figure 1e was analyzed using a linear mixed-effects model with time (Week) × Group interaction as fixed effects and mouseID as a random intercept. Pairwise comparisons of tumor growth rates were performed using estimated marginal trends with Tukey correction for multiple comparisons. The analysis was conducted in R using the *lme4* and *emmeans* packages. A *P* value less than.05 was considered statistically significant. Data are presented as mean ± SD, except for tumor volumes in Figure 1e that are only presented as + SD.

## Ethics Statement

Mice were bred at the Department of Experimental Medicine, University of Copenhagen and transported to Department of Dermatology, Copenhagen University Hospital—Bispebjerg Hospital. The performed animal studies were approved by the Danish Animal Experiments Inspectorate (protocol 2019-15-0201-01666) in accordance with Directive 2010/63/EU and the Animal Welfare committee at Copenhagen University Hospital—Bispebjerg Hospital.

Human tissue sample was obtained following written informed consent from the participant. The study was reviewed and approved by Danish Research Ethics Committee, Region Hovedstaden (H-20009236).

## Data availability statement

The RNA sequencing data generated in this study are deposited in the Gene Expression Omnibus under accession number GSE311891 (https://www.ncbi.nlm.nih.gov/geo/query/acc.cgi?acc=GSE311891). All data analysis scripts are documented in an R Markdown file and are available at: https://doi.org/10.5281/zenodo.17593648.

## Conflict of Interest

The authors state no conflict of interest.
